# A Bibliometric Analysis of Platelet-Rich Fibrin in Dentistry

**DOI:** 10.3390/ijerph191912545

**Published:** 2022-10-01

**Authors:** Hsin-Ying Yu, Yu-Chao Chang

**Affiliations:** 1 School of Dentistry, Chung Shan Medical University, Taichung 40201, Taiwan; 2 Department of Dentistry, Chung Shan Medical University Hospital, Taichung 40201, Taiwan

**Keywords:** platelet-rich fibrin, bibliometric analysis, dentistry, oral surgery, wound healing, growth factors

## Abstract

Platelet-rich fibrin (PRF), a second-generation platelet concentrate, has been widely recognized in numerous studies for its performance of wound healing and regeneration in dentistry. However, bibliometric analysis of PRF in dentistry is still scarce. Thus, this study aimed to conduct and delineate a bibliometric analysis of the application of PRF and its changing trend in dentistry. All papers concerning PRF up to 30 June 2022 were included in the literature search from the Web of Science Core Collection database. These data were then entered into Microsoft Excel, analyzed by the SAS statistical software, and visualized by the VOSviewer software. A total of 562 articles were retrieved following the exclusive criteria. The results demonstrated that the trend of annual publication increased continually (*p* for trend < 0.01), more notably in the last five years. The majority of the articles were original (58.01%), followed by reviews (17.08%), and case reports (10.14%). The three major study designs were clinical studies (20.11%), randomized controlled trials (17.62%), and review/meta-analysis (17.08%). PRF was most frequently employed in oral surgery (31.14%), periodontal regeneration (22.42%), and implant therapy (18.68%). Turkey (16.19%), India (12.28%) and China (7.12%) were the top 3 countries publishing PRF studies. By comparing the total number of publications, total citation counts and varying counting methods, a combination of numerous counting methods was suggested for use since each counting method yields different results. Taken together, we hope with these informative findings, researchers could focus on the future direction or advancement in PRF, laying a foundation for evidence-based dentistry.

## 1. Introduction

Platelet-rich fibrin (PRF), a second-generation platelet concentrate described by Choukroun et al. [1], is an autologous fibrin matrix rich in platelets and growth factors without the addition of anticoagulant or bovine thrombin during preparation [2]. The fibrin network of PRF could facilitate cell migration, proliferation, and differentiation [3,4]. This distinctive structure may be considered a vehicle for transporting cells that are crucial for tissue regeneration.

Potential clinical indications of PRF in clinical dentistry are diverse and copious, such as healing in extraction sockets [5,6], grafting material in sinus floor augmentation before implant placement [7], regeneration of soft tissue periodontal defects [8,9,10], management of young immature necrotic permanent teeth [11,12], and tissue healing in various oral pathologies, such as medication-related osteonecrosis of the jaw (MRONJ) [13,14]. Its healing and regenerative capacity have increasingly drawn attention worldwide; as shown in the abundant systematic reviews and meta-analyses [15,16,17]. However, to our knowledge, the essential information concerning the developing trends of PRF application in dentistry with a macroscopic view was limited.

To provide a guide to recognize rising and promising studies, a bibliometric analysis could offer a macro-perspective measure to perform a mathematical and statistical analysis for voluminous information. This robust study tool evaluates the impacts of scientific research and the performance of individuals, journals, publications, institutes, and country correlations in a specific field, but with an extensive database [18,19,20,21]. Unlike the systematic literature review summarizing the discoveries of existing literature or meta-analysis focusing on the results among homogenous studies, bibliometric analysis deciphering and data mapping the state of the intellectual constitution and developing trends of a study field over time [22].

A bibliometric analysis combines both objective indexes, such as citation counts, publishing patterns, frequent occurrence of keywords, number of published papers worldwide, and the amount of global scientific cooperation, as well as subjective indexes, including the dominant topic of interests and thematic analysis [18]. One of the frequently used bibliometric visualization software, the VOSviewer (Leiden University, Leiden, The Netherlands), facilitates analyzing the strength of links between prolific authors, journals, institutions, and nations, as well as popular keywords with graphical representations in an easy-to-interpret way. By evaluating the indexes above and depicting them with VOSviewer, we can construct a comprehensive analysis of bibliometric diagrams, which can aid in orienting experimentation strategies and funding decisions [20,23].

Even though the bibliometric studies are becoming more popular in current research fields, little is known about the bibliometric analysis of PRF in dentistry. Therefore, this study aimed to conduct and delineate a bibliometric analysis of PRF application and its changing trend in dentistry, in the hope of paving the way for future research, established on the hotspots or emerging topics over the last two decades.

## 2. Materials and Methods

### 2.1. Database and Search Strategy Selection

**Figure 1:**
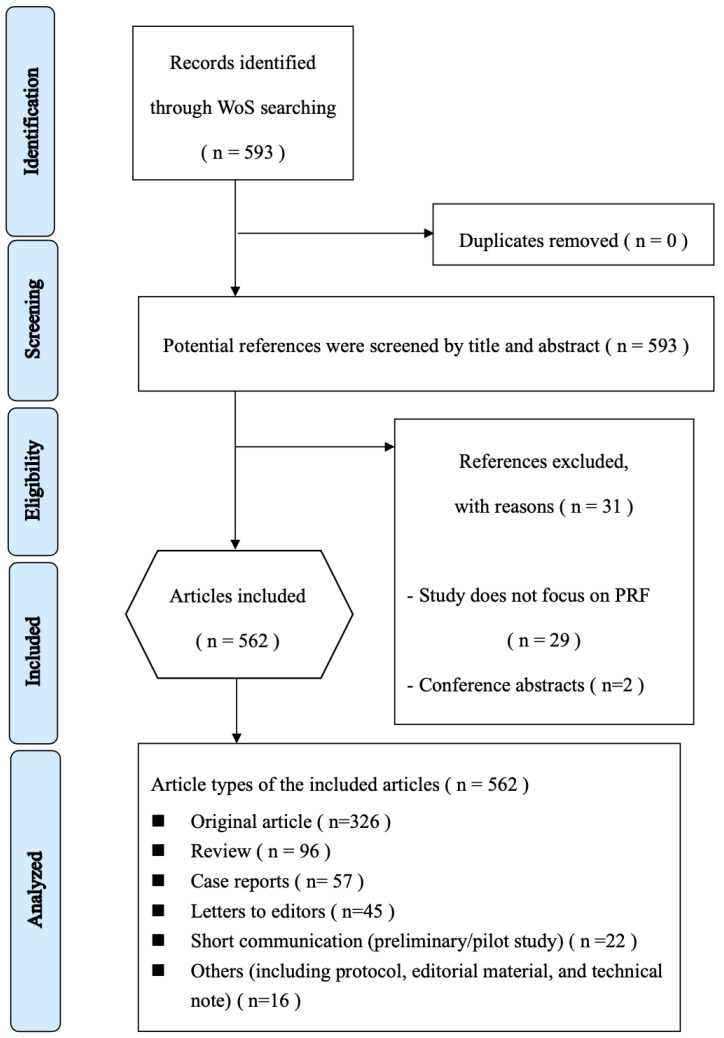
PRISMA selection strategy flow diagram.

Web of Science (WoS) is the oldest citation database and has the most comprehensive records of literature with detailed citation analysis [24]. In this study, both Science Citation Index Expanded (SCIE) and the Social Science Citation Index (SSCI) databases are used in WoS on 31 June 2022. The search strategy was Topic = (“platelet rich fibrin”) OR Topic= (“platelet-rich fibrin”) OR Topic = (“PRF”) in “Dentistry Oral Surgery Medicine” field. Two reviewers independently screen titles, abstracts, and contents, of potential references regarding PRF in dentistry. The exclusion criteria were applied to the following conditions: (1) not associated with PRF; (2) a conference abstract. In case of disagreement between reviewers on inclusion and exclusion criteria, a consensus was resolved after discussion. After the process of exclusion, the bibliometric data of the included references were exported from WoS for further investigation. The selection strategy is presented as PRISMA flow diagram and illustrated in Figure 1.

### 2.2. Bibliometric Analysis

The objective indexes (number of articles, authors, affiliations, study design and its subdivision, source of journals, citation counts, geographical distribution) and subjective indexes (scope category of research, keywords, and the main function of PRF in the article) were recorded in Microsoft Excel for descriptive bibliometric analysis. The geographical distribution was recorded from the address of correspondence author. Corresponding authors were recorded for authorship productivity, all authors in every article were recorded for author’s relationship and all the author’s affiliations of origin were noted. Therefore, the number of authors and institutes of origin were more than the number of articles included. In case of discrepancy in the interpretation of research, a consensus was reached with the second author after discussion.

#### 2.2.1. Manuscript Type and Study Design

Concerning the type of manuscript, the following six categorical variables categorized as original, review, case reports, short communication (including preliminary study and pilot study), letters to editors, and others (including protocol, editorial material, and technical note) according to the arrangement from their journals.

Regarding the study design, the following eight subdivisions of study type were catalogued: clinical study; randomized controlled trial (RCT); review and meta-analysis; case reports; animal study; in vitro research; and pilot/preliminary study. Articles combining animal study and in vitro research were included in the animal study category.

#### 2.2.2. Scope Category of Research

Regarding the scope category of an article, each article was classified into one of the seven fields in dentistry by the content, which were oral surgery, periodontics, implantology, oral biology, endodontics, orthodontics, and oral pathology.

### 2.3. Statistical Analysis

To ensure the reliability and validity, all the extracted articles were conducted and analyzed three times by Dr. Yu. In case of discrepancy in the interpretation of the study, a consensus was reached with the second author after discussion. The kappa value for intra-rater reliability was 0.9031, which was a strong level of agreement. After the collection of bibliometric data, they were exported to the SAS version 9.1 statistical software (SAS Institute, Cary, NC, USA) to perform the Fleiss’ Kappa Statistics for intra-rater reliability and Pearson’s chi-square test. Probability levels of <0.05 were considered significant. On the other hand, the VOSviewer version 1.6.17 (Leiden University’s Centre for Science and Technology Studies, Leiden, Netherlands) (https://www.vosviewer.com/, accessed on 30 June 2022), developed by Nees Jan van Eck and Ludo Waltman, was employed to depict a bibliometric network map of co-authorship, co-citation authors, countries, affiliations, and keywords [23].

## 3. Result

### 3.1. Article Types, Designs and Scope Category

**Figure 2:**
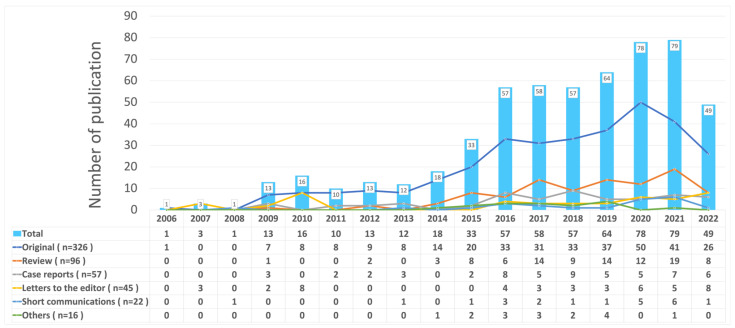
The annual production of different article types in PRF studies.

A total of 562 articles were identified using the search strategy in the WoS database; after screening the titles, abstracts and were included for further bibliometric analysis. A total of 31 articles were excluded for the following reasons: not regarding PRF (n = 29); conference abstracts (n = 2). As Figure 2 depicts, the trend of annual publication increased continually until the data collection in 2021 (*p* < 0.01) and grew noticeably in the last five years. The most frequently published article type was original articles (n = 326, 58.01%), followed by review (n = 96, 17.08%), and case reports (n = 57, 10.14%). The major study designs were clinical study (n = 113, 20.11%), randomized controlled trials (n = 99, 17.62%), review and meta-analysis (n = 96, 17.08%), in vitro research (n = 58, 10.32%), case reports (n = 57, 10.14%), and animal study (n = 56, 9.96%).

**Figure 3:**
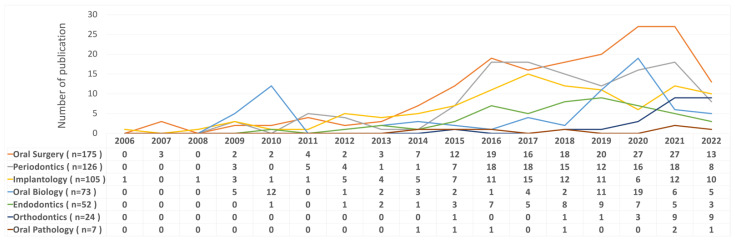
The annual production of PRF studies in different scope categories in dentistry.

The classification of scope category was carried out by Dr. Yu three times. In case of discrepancy in the interpretation of the study, a consensus was reached with the second author after discussion. The kappa value for intra-rater reliability was 0.9031, which was a strong level of agreement. As Figure 3 illustrates, the applications of PRF were most used in oral surgery (n = 175, 31.14%), periodontal regeneration (n = 126, 22.42%), and implant therapy (n = 105, 18.68%). Additionally, it reveals the annual production of PRF studies in different scope categories in dentistry. PRF was mostly placed in the extraction socket (n = 70, 12.99%), sinus lift (n = 47, 8.72%), intrabony defects (n = 41, 7.61%) for tissue engineering, soft tissue for gingival recession (n = 31, 5.75%), as well as the human’s root canal (n = 27, 5.01%) for regenerative endodontic treatment. The function of PRF were mostly for tissue engineering (n = 426, 75.8%), regenerative endodontic treatment (n = 39, 6.94%), and the manipulation and characterization of PRF was also frequently studied (n = 36, 6.94%).

### 3.2. Authors, Affiliations and Country

**Figure 4:**
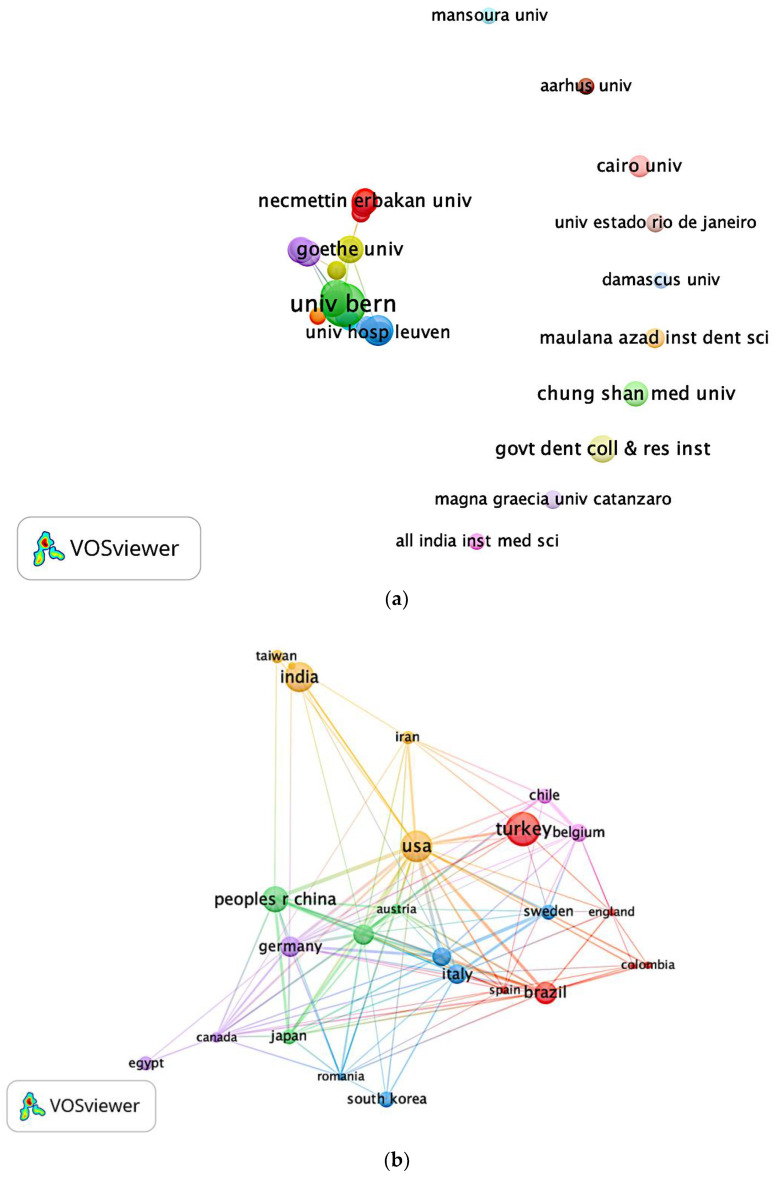
Bibliometric analysis of (**a**) Affiliation network map, and (**b**) Country network map. Different colors denote various clusters, and the size of the circles represents the quantity of publications. The distance between the two circles reveals the relatedness between them.

Regarding the authorship productivity and total citation counts, the most frequently published corresponding authors were: Richard J. Miron (n = 18, 897 citations), David M. Dohan (n = 16, 2258 citations), Yu-Chao Chang (n = 11, 320 citations), Shahram Ghanaati (n = 9, 348 citations), and Avani Raju Pradeep (n = 9, 300 citations). In relation to the authors’ affiliations, the University of Bern contributed the maximum number of articles with 36 PRF related studies, followed by the University of Wuhan (n = 19), Katholieke Universiteit Leuven (n = 17), and Goethe University, Frankfurt (n = 14). The co-authorship network of affiliations published at a minimum of five articles is illustrated in Figure 4a.

**Figure 5:**
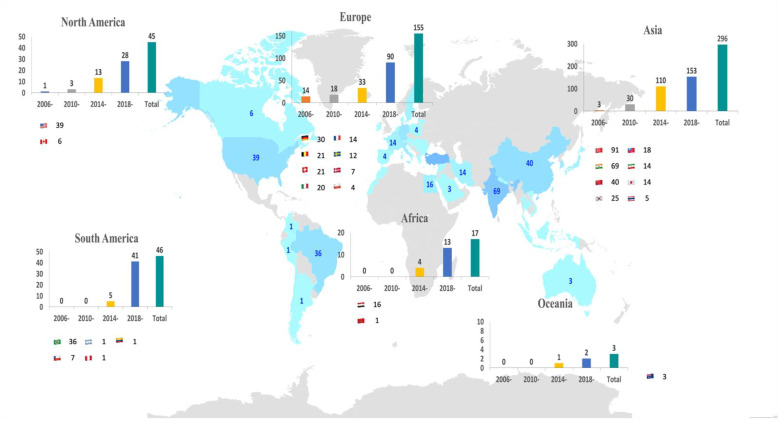
The current publications of PRF studies worldwide according to corresponding author. The blue color highlighted on the map represents the countries which have published PRF-related studies. The more the navy blue is the higher number of publication that the country has published.

Concerning the country productivity, the co-authorship network of countries is depicted in Figure 4b. With the corresponding author’s origin counting method being used, Asia (n = 296, 52.67%), Europe (n = 155, 27.58%), and South America (n = 46, 8.19%), were the three major continents which published the highest total number of articles in the world. As Figure 5. demonstrates, the five main countries contributing the highest total number of publications were Turkey (n = 91, 16.19%), India (n = 69, 12.28%), China (n = 40, 7.12%), the United States (USA) (n = 39, 6.94%), and Brazil (n = 36, 6.41%). It is worthwhile noticing that if all co-authors were counted by full counting method, the results were quite different from corresponding author counting. The five main countries contributing the highest total number of publications were Turkey (n = 95), USA (n = 81), India (n = 72), China (n = 59), and Brazil (n = 44).

### 3.3. Journal

The Journal of Periodontology published the largest number of articles (n = 51, 9.07%) for PRF, followed by Clinical Oral Investigation (n = 49, 8.72%), and Journal of Oral and Maxillofacial Surgery (n = 33, 5.87%). From the top 10 journals with the most publications, the oral surgery related journals, including Journal of Oral and Maxillofacial Surgery, Journal of Cranio-maxillofacial Surgery, and International Journal of Oral and Maxillofacial Surgery, constituted the largest number of articles (n = 83). While the periodontology related journals, including the Journal of Periodontology and International Journal of Periodontics and Restorative Dentistry, published 72 articles, as well as implant related journals, International Journal of Oral and Maxillofacial Implants and Journal of Oral Implantology, which published 40 articles. Table 1 lists the most productive journals with PRF related publications in WoS journals. These journals published around one third of PRF articles (34.7%) and made up the core sources of PRF research.

### 3.4. Citation Count

**Figure 6:**
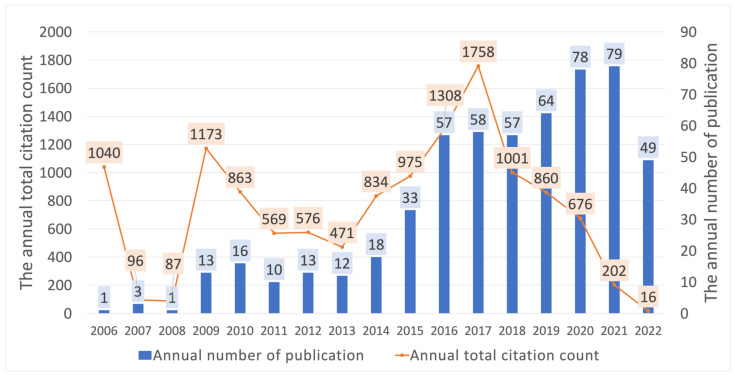
The annual total citation count and production of PRF studies.

The number of PRF studies each year increased progressively over time, which is displayed in Figure 6. The total citation counts reached three climaxes in 2006, 2009, and the highest in 2017; thereafter, they dropped dramatically the following year. The article [25] from Choukroun et al., in 2006, which was recorded as the first collection in this bibliometric data, has earned the highest total citation count (1040) in WoS until now. On the other hand, the study by He et al. [26], Kobayashi et al. [27], and Elgali et al. [28], contributed the majority of total citation counts in 2009, 2016, and 2017, in WoS, respectively. The top 20 cited articles are enumerated in Table 2 according to the total number of citations in descending order.

### 3.5. Keyword

**Figure 7:**
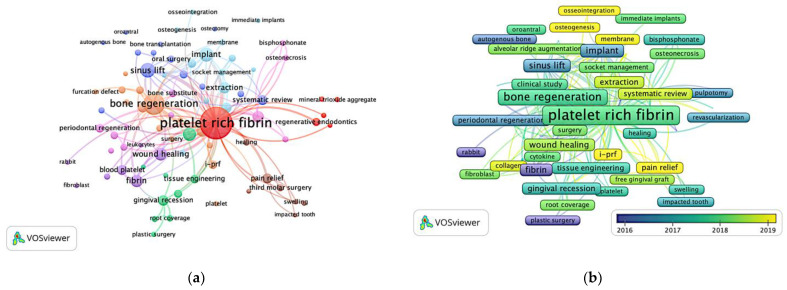
Bibliometric analysis of author keyword: (**a**) Eight trend topics of PRF studies, which were bone regeneration in orange, wound healing in purple, periodontology in green, molar surgery and pain relief in brown, regenerative endodontics in red, as well as implant and osseointegration in light blue; (**b**) Yearly distribution of overlay author keyword visualization after normalization. It showed that more research pertained on PRF derivates, a-PRF and i-PRF, as well as pain relief, osseointegration, and systematic review, have been published recently.

There were eight themes identified in the PRF studies, as shown in Figure 7a, illustrated by the VOSviewer. The orange cluster included studies related to the topic of bone regeneration. In the pink cluster, periodontal regeneration and osteonecrosis were the subject of research. Wound healing was part of the purple cluster. The green cluster included studies looking at periodontology field, such as gingival recession, root coverage, and plastic surgery. Studies looking into third molar surgery and pain relief were included in the brown cluster. The red cluster represented the regenerative endodontics domain. Studies investigating implant and osseointegration were included in the light blue cluster, while studies discussing sinus lift and bone transplantation were part of the dark blue cluster. Figure 7b is portrayed by the VOSviewer, which convey the information that more research pertained on PRF derivates, a-PRF and i-PRF, as well as pain relief, osseointegration and systematic review, have been published recently.

## 4. Discussion

Our study has demonstrated an increasing trend in the number of publications on PRF research in the last decade. From 2006 to 2014, the annual number of published articles increased steadily and has risen drastically since 2015. This upward trend could be due to the expanding favorable regeneration potential of PRF, in addition to the increased awareness and acceptance of PRF usage among dental professionals. The number of articles in original form and review also presented a growing tendency, which is not surprising since PRF has been the focus of attention around the world since Choukroun et al. [1,25] revealed the regenerative capacity of PRF. Even until now, PRF is still a trending topic owing to the observation that derivatives of PRF, such as leucocyte-poor or pure platelet-rich fibrin (P-PRF), leukocyte and platelet-rich fibrin (L-PRF), advanced PRF (A-PRF), and injectable PRF (i-PRF), which resulted from different manipulation but possessed various functions and indications. Therefore, this may explain why publications regarding PRF are still rising noticeably at present.

Regarding PRF application in different scope categories in dentistry, the number of studies in oral surgery, periodontics, and implantology, remained the majority of all in both manual classification and journal distribution in our study. One review [29] also showed that oral-maxillofacial surgery and periodontics accounted for the primary contribution to PRF studies. Currently, there are numerous studies on the use of PRF in mandibular third molar surgery for preventing dry sockets during the first 7 days [30,31,32]. This explains why the extraction socket is the most frequent location for PRF placement in our study. Followed by extraction sockets in oral surgery treatments, sinus lifts ranked the second most frequent therapy involving PRF. The selection of sinus lift procedures is highly correlated with implant placement because sinus lift related articles were designated in the implant category in our study. Since the growth factors were secreted from PRF inducing angiogenesis and boosting blood flow into the sinus cavity, this favored and accelerated bone healing and the further osteointegration in implant treatment [30]. Concerning the application of PRF in periodontics, intrabony defects and gingival recession were the third and fourth primary placements in our study, respectively. Owing to PRF’s abilities to accelerate the healing of tissues as well as reducing pain and inflammation, PRF provides access to a multitude of regenerative therapies [15].

Most studies related to platelet-rich plasma (PRP) or periodontal regeneration showed that the United States is the most productive country in dentistry [33,34]. Surprisingly, the results in our study showed that Turkey is the most productive country in PRF studies, as a result of the first corresponding author counting method; one possible reason is the application of different counting methods used for geographical distribution. With the VOSviewer counting method, if full counting is used, each co-author is given full credit for every publication. An article, for instance, that was co-authored by four nations counts as a full publication for each of the four nations [35]. In our study, even if an article that was co-authored by four nations counts as a full publication for only one nation, that is of the first corresponding author. The weight for the other co-authors’ nationalities is zero. This counting method was adopted by Moya-Anegón et al. [36] who stated that the concept that the publication’s corresponding author might be viewed as the research guarantor is what encourages corresponding author counting. An interesting result was found that when all co-authors were counted, the United States moved up from fourth (n = 39) to second place (n = 81). This fact indicates that there was a great deal of international collaboration involving authors from the United States. Therefore, a combination of numerous counting methods was suggested for use since each counting method yields different results [37]. In this way, the authors avoided bias and provided a comprehensive view of bibliometric analysis. Additionally, the VOSviewer ought to be optimized by way of different counting methods of authors, such as corresponding author and first author.

In terms of the most active authors, they are usually highly related to the most productive affiliations. In our study, the most frequently published corresponding author was Richard J. Miron, who also comes from the most productive institution, the University of Bern. Nonetheless, some authors have established their research activity in different affiliations during their lifetime. For instance, one of the most prolific authors, David M. Dohan Ehrenfest, conducted his research activity in various institutions, UDICE-French Research Universities (France), Universite de Paris (France), University of Gothenburg (Sweden), and Chonnam National University (South Korea). This result points out that extreme caution should be taken while organizing enormous bibliometric data. Another finding that deserves mention is that Richard J. Miron also appeared to have the strongest network of collaboration with other prolific authors. Collaboration is crucial since it increases interaction with specialists from various fields and increases the chance of funding, as well as the wider readership [38].

Citation analysis can be used to uncover the interdisciplinary or multidisciplinary nature of research initiatives and programs, as well as to establish fields and developing specialties through journal relationships [39]. In our study, the annual total citation counts spiked in 2006, 2009, and 2017, respectively. The study by Choukroun et al. [25] in 2006 clearly has the highest total number of citations of any PRF study in WoS and is expected to continue to receive citations in the future. As it is the first study that tested and described PRF, researchers who focus on PRF obviously cite this pioneering work. Another two peaks in total citation count are shown in 2009 and 2017, where we can observe that the top cited articles were no longer original articles; but rather reviews. It is predictable that original articles received higher citation counts owing to their novelty and the lack of studies at that time. After several years of scientific and clinical improvement and the accumulation of high-quality studies, review articles were published and recognized by an increasing number of scholars. Another fact to be noticed is that although Turkey published the highest total number of articles in the world, there is no article from Turkey listed in the 20 top-cited articles (see Table 2). A possible explanation is that there is less collaboration in Turkey and therefore less readership than in the United States and in Western Europe, which reflected the importance of international teamwork proposed by Barão et al. [38] Reliability-related concerns resulting from the perceived selectivity and limitedness (e.g., language and region) were also present [40]. These results suggested that larger publications from a country, an institution, a journal, or an author, do not completely correlate with higher quality of the articles. Donthu et al. [21] believed that when drawing conclusions from bibliometric data, researchers should exercise extreme caution and support their claims with content analysis.

From the co-occurrence keyword network, we can see that the most common keyword “PRF” is strongly associated with “bone regeneration” and “wound healing”, which are obviously the main functions of PRF. The mechanism of growth factors in repair has attracted a considerable amount of intense discussion, similar to another bibliometric analysis [33] investigating PRP application in orthopedics. The trending topic of PRF usage includes management of sinus lift and extraction socket for oral surgery treatment [41,42,43], osseointegration and alveolar ridge preservation for further implant therapy [44], gingival recession and root coverage for periodontal treatment [9], and mineral trioxide aggregate combined with PRF for regenerative endodontic treatment [45]. After further chronological analysis, pain relief with PRF in pulpitis [46], and third molar surgery [47], were found to be effective and popular in the recent 10 years. Additionally, PRF derivates (a-PRF and i-PRF) with different manipulation techniques showed a better result in healing and releasing higher concentrations of various growth factors [48,49]. However, a larger sample and a longer follow up in clinical practice are encouraged to verify their effectiveness.

This study applies bibliometric methods for depicting the development of PRF in dentistry; nonetheless, there are some limitations that must be addressed. Firstly, only one database (WoS) was adopted, hence several articles that were relevant to PRF would have been omitted. Secondly, although there is no language restriction in our study, the majority of WoS publications are in English, which may generate linguistic prejudice. Thirdly, the lack of self-citation correction by a journal or author may result in an increase in the number of self-citings, which could lead to mistakes in the analysis. Authors occasionally need to cite their previously published papers in a study series, even though it is never recommended to engage in the improper practice of self-reference to boost the number of citations for the publication. Lastly, although citation analysis has been implemented in this study, a combination of quality assessments would be preferable to reflect truly high-quality articles. Unlike systematic review and meta-analysis, which only include high standard articles, bibliometric analysis is intended to offer a comprehensive insight into a subject of interest. Notwithstanding the limitations, this study still delivers a helpful perspective on the advancements and evolving trends in PRF in dentistry over the past two decades.

## 5. Conclusions

To the best of our knowledge, this is the first study to analyze bibliometric data of PRF studies in dentistry. According to the result presented, the studies of PRF have escalated annually. In particular, the study design focused on clinical studies and randomized controlled trials, laying the foundation for evidence-based dentistry. Furthermore, combining different counting methods is crucial to provide a fully integrated map of interest to avoid prejudice and a limited view of bibliometric analysis.

## Figures and Tables

**Table 1:** Most productive journals with PRF related publications in WoS (from Journal Citation Reports^TM^ 2021) (position in dentistry, oral surgery and medicine).

Journal	Q	Position	IF	Country Edition	Total Docs	Total Cites	Average Cites/Docs
Journal of Periodontology	1	12/92	4.494	USA	51	1741	34.14
Clinical Oral Investigations	2	25/92	3.606	Germany	49	1054	21.51
Journal of Oral and Maxillofacial Surgery	4	73/92	2.136	UK	33	521	15.79
Journal of Endodontics	1	13/92	4.422	USA	28	570	20.36
Journal of Cranio-Maxillofacial Surgery	2	34/92	3.192	UK	27	534	19.78
International Journal of Oral and Maxillofacial Surgery	2	40/92	2.986	USA	23	269	11.70
Implant Dentistry	2	39/92	3	USA	21	591	28.14
International Journal of Periodontics & Restorative Dentistry	4	70/92	2.227	USA	21	201	9.57
International Journal of Oral & Maxillo-facial Implants	2	44/92	2.912	USA	20	279	13.95
Journal of Oral Implantology	4	85/92	1.546	USA	20	600	30.00
Oral Surgery, Oral Medicine, Oral Pathology and Oral Radiology	3	56/92	2.538	USA	19	2070	108.95

**Table 2:** Top 20 most-cited articles related with PRF in WoS.

Rank	Article Title	First Author	Year of Publication	Article Type	Country	Times Cited	Reference
1	Platelet-rich fibrin (PRF): A second-generation platelet concentrate Part V: Histologic evaluations of PRF effects on bone allograft maturation in sinus lift	Choukroun, J.	2006	Original	France	1040	https://doi.org/10.1016/j.tripleo.2005.07.012
2	A comparative study of platelet-rich fibrin (PRF) and platelet-rich plasma (PRP) on the effect of proliferation and differentiation of rat osteoblasts in vitro	He, Ling	2009	Original	China	276	https://doi.org/10.1016/j.tripleo.2009.06.044
3	Comparative release of growth factors from PRP, PRF, and advanced-PRF	Kobayashi, Eizaburo	2016	Original	Switzerland	250	https://doi.org/10.1007/s00784-016-1719-1
4	Guided bone regeneration: materials and biological mechanisms revisited	Elgali, Ibrahim	2017	Review	Sweden	230	https://doi.org/10.1111/eos.12364
5	Three-Dimensional Architecture and Cell Composition of a Choukroun’s Platelet-Rich Fibrin Clot and Membrane	Ehrenfest, David M. Dohan	2010	Original	Sweden	225	https://doi.org/10.1902/jop.2009.090531
6	Advanced Platelet-Rich Fibrin: A New Concept for Cell-Based Tissue Engineering by Means of Inflammatory Cells	Ghanaati, Shahram	2014	Original	Germany	225	https://doi.org/10.1563/aaid-joi-D-14-00138
7	Mineral trioxide aggregate and other bioactive endodontic cements: an updated overview—part I: vital pulp therapy	Parirokh, M.	2018	Review	Iran	165	https://doi.org/10.1111/iej.12841
8	Sinus Floor Augmentation With Simultaneous Implant Placement Using Choukroun’s Platelet-Rich Fibrin as the Sole Grafting Material: A Radiologic and Histologic Study at 6 Months	Mazor, Ziv	2009	Case reports	Sweden	164	https://doi.org/10.1902/jop.2009.090252
9	Use of platelet-rich fibrin in regenerative dentistry: a systematic review	Miron, Richard J.	2017	Review	Switzerland	160	https://doi.org/10.1007/s00784-017-2133-z
10	Clinical Evaluation of a Modified Coronally Advanced Flap Alone or in Combination With a Platelet-Rich Fibrin Membrane for the Treatment of Adjacent Multiple Gingival Recessions: A 6-Month Study	Aroca, Sofia	2009	Original	France	150	https://doi.org/10.1902/jop.2009.080253
11	Optimized Platelet-Rich Fibrin With the Low-Speed Concept: Growth Factor Release, Biocompatibility, and Cellular Response	Fujioka-Kobayashi, Masako	2017	Original	Switzerland	150	https://doi.org/10.1902/jop.2016.160443
12	In vitro effects of Choukroun’s PRF (platelet-rich fibrin) on human gingival fibroblasts, dermal prekeratinocytes, preadipocytes, and maxillofacial osteoblasts in primary cultures	Ehrenfest, David M. Dohan	2009	Original	Sweden	142	https://doi.org/10.1016/j.tripleo.2009.04.020
13	Injectable platelet rich fibrin (i-PRF): opportunities in regenerative dentistry?	Miron, Richard J.	2017	Original	Switzerland	121	https://doi.org/10.1007/s00784-017-2063-9
14	Choukroun’s platelet-rich fibrin (PRF) stimulates in vitro proliferation and differentiation of human oral bone mesenchymal stem cell in a dose-dependent way	Ehrenfest, David M. Dohan	2010	Original	Sweden	110	https://doi.org/10.1016/j.archoralbio.2010.01.004
15	Regenerative endodontics: a comprehensive review	Kim, S.G.	2018	Review	Australia	109	https://doi.org/10.1111/iej.12954
16	Regenerative potential of leucocyte- and platelet-rich fibrin Part A: intra-bony defects, furcation defects and periodontal plastic surgery A systematic review and meta-analysis	Castro, Ana B.	2017	Review	Belgium	107	https://doi.org/10.1111/jcpe.12643
17	Simultaneous Sinus-Lift and Implantation Using Microthreaded Implants and Leukocyte- and Platelet-Rich Fibrin as Sole Grafting Material: A Six-Year Experience	Simonpieri, Alain	2011	Original	Sweden	106	https://doi.org/10.1097/ID.0b013e3181faa8af
18	Autologous Platelet-Rich Fibrin in the Treatment of Mandibular Degree II Furcation Defects: A Randomized Clinical Trial	Sharma, Anuj	2011	Original	India	104	https://doi.org/10.1902/jop.2011.100731
19	Effects of Choukroun’s platelet-rich fibrin on bone regeneration in combination with deproteinized bovine bone mineral in maxillary sinus augmentation: A histological and histomorphometric study	Zhang, Yu	2012	Original	Austria	103	https://doi.org/10.1016/j.jcms.2011.04.020
20	Platelet-rich fibrin membranes as scaffolds for periosteal tissue engineering	Gassling, Volker	2010	Original	Germany	100	https://doi.org/10.1111/j.1600-0501.2009.01900.x

## Data Availability

The data presented in this study are available on request from the corresponding author.
